# Remarks on Croup

**Published:** 1838-12-05

**Authors:** C. D. Meigs


### REMARKS ON CROUP. By C. D. Meigs, M.D.

To the Editors of the Medical Examiner:

*Gentlemen,*—For more than twenty-five years I have had very numerous opportunities of observing the disease called croup, and vulgarly known as hives. In my experience, I have not frequently seen the malady terminate fatally, except when complicated with scarlatina. I have so generally found it to yield readily to treatment of a mild kind, that I have suspected a major part of the cases to be unattended with active inflammation, and hence to be far less dangerous than commonly supposed; and less frequently demanding for their cure, a resort to venesection and harsh measures, such as leeching, blistering, &c., than is usually imagined. It is on this account that I feel prompted to send you this essay, with a hope that it may not be altogether inopportune at a season when the anginose disorders begin to be prevalent.

Cynanche trachealis, or croup, consists in inflammation or irritation of the mucous lining of the larynx. Inflammation of this tissue, is liable to one of the common terminations, or cures of inflammation, to wit, the effusion of a fluid. It is not inevitable, in every case, for the cure to be effected by effusion. The disorder may go off by resolution, which is the promptest and safest result, and one which should always be procured, if possible.

When effusion does take place, it may consist of a mucus containing a very large proportion of albumen, and so coherent to the surface from which it exudes that it seems to be really plastic; and does, in fact, assume the form of an artificial membrane, lining the larynx and trachea, and becoming thicker with every additional drop effused, so that at length the admission of air to the lungs is almost wholly prevented, and the patient dies with asphyxia.

It does not happen in every case that the effusion is followed by such unhappy consequences, for from the vessels of the larynx may issue a considerable quantity of mucus so very liquid that it keeps up a constant cough and screatus, with feeling of soreness in the windpipe, and, as the liquid is expectorated as fast as it is effused, no considerable angina is occasion by the disorder. In all cases of laryngeal inflammation, with tendency to plastic effusion, the exigency of the demand for relief is very great, as the patient is hurried with rapid strides to a fatal issue. The lymph adheres so firmly that no effort of the patient, either in coughing or vomiting, can be reasonably expected to detach it. If the physician does not arrive in time to prevent the disastrous tendency to effusion of lymph, his subsequent endeavours will, for the most part, prove unavailing. But, it is consolatory to reflect, that, in some few instances, life may be preserved after the most unequivocal evidences have been had of the existence of a plastic secretion in the windpipe.

The croup that proves fatal, so far as my observations enable me to speak with confidence, does not attack suddenly. The inflammatory turgescence of the membrane progresses slowly, and is marked by occasional cough; yet more particularly by hoarseness of the voice, which gradually looses its sonorousness, and sinks into a whisper, in speaking or crying; but gives out the trumpet, or croup sound, in coughing, and only then. This whispering of the voice, sometimes continues a day or two before the sound of croup can be heard, and when it is heard, the danger is already great, and the catastrophe imminent.

In another, and far more common, form of the malady, the patient is attacked without any premonitory signs. If it be a child, he has, probably, been put to bed in apparent health, and after sleeping an hour, or more, is seen to start suddenly from sleep, with all the appearances of a person strangling. The croup sound is heard at once, and of the loudest tone. The child struggles, both from alarm and from a sense of suffocation; but after three or four minutes begins to be more composed, yet coughing frequently, with the sound peculiar to the case. The fright, and the struggle, generally occasion a disturbance of the circulation. The pulse becomes frequent and full, but the temperature of the body is not augmented, and, in a short time, it is found there is no fever. A mild remedy is employed, the patient soon falls asleep, and has no return of the croup during the remainder of the night. It is highly probable, however, that he will have a similar, but milder attack in the following night, after which he is well.

Such, I am confident, is a just picture of a great majority of the cases of croup. These cases prove very rarely fatal, and are easily cured without resort to harsh remedies, and without the necessity of doing anything that may affect the child's health for a moment after the principal symptom is vanquished.

From the foregoing remarks, it is apparent that I suppose the existence of two forms of the disease,—one highly inflammatory and exceedingly dangerous, the other far less inflammatory and scarcely furnishing fit occasion for a moment of anxiety. In the former there is the strongest tendency, from a slowly increased inflammation, to the effusion of plastic lymph; in the latter there is inflammatory irritation of the larynx, with tendency not to effusion, but to the induction of spasm of the glottis. The one is an acute inflammation, the other is a spasmodic affection, the result of irritation in the windpipe.

I think this view of the case is enforced by a consideration of the effects of remedies. In the dangerous croup, which is cured by venesection, the croup sound continues for hours; abating by degrees only, and only by means of the most copious depletion; in the latter, the croup sound often ceases entirely, and never returns, upon the exhibition of a small quantity of ipecacuanha, or any other emetic substance, even where no emesis is produced. I am inclined to think that, in the spasmodic form, the sound is more distressing than in the more dangerous case,—for I have always observed that, as the dangerous case advances to its fatal conclusion, the croup sound ceases to be heard, the voice being no longer sonorous, as it is uttered by a tube lined with soft phlegm and lymph. It is probable that most of your readers will concur with me as to the justness of this observation. Again, it is to be remarked, that, in the fatal form of croup, there is very little or no remission of the peculiar sound of the cough, until the power of phonation is lost in the total alteration of the laryngeal surface; whereas, in the spasmodic form, the child croups violently for a few minutes, and then ceases altogether to do so; or he ceases for a little while, breathing easily, and without wheezing; after which, he may be seized again with the spasm, so as to produce all the phenomena in their greatest intensity, again and again, in the course of a night,—and appearing in the morning to be either quite well, or suffering only from a slight hoarseness and soreness of the throat.

Now, I admit that it is necessary to make a careful diagnosis of these two forms of the disease, inasmuch as the most fatal consequences might follow the mistaking of the one case for the other; or the child might be obliged to endure a very harsh treatment which, besides being wholly unnecessary, would be cruelty to no purpose.

The discrimination is to be made by a careful examination of the pulse, the state of the skin, the respiration, and symptoms, which existed up to the time of calling in the physician. If the pulse be not much disturbed, or not more than can be well attributed to the alarm and agitation of the patient, if the skin be not of an increased temperature, if the respiration be not all the time difficult, and if the attack shall have been sudden and without premonition, then a foot-bath with mustard, and an emetic of ipecacuanha, is in general all that is necessary for the cure. A small teaspoonful of the ipecacuanha may be mixed with a wineglassful of water, and given in doses of a teaspoonful of the mixture every ten, fifteen, or twenty minutes, according to the urgency of the symptoms. I have seen many apparently severe attacks yield to a single dose of the mixture, and without emesis; and very numerous instances in which a single act of vomiting has sufficed to dispel all the anxiety of the child's friends, by the complete relief which has followed the operation.

An emetic is incapable of curing the more violent or inflammatory croup, and notwithstanding the very considerable relief which is procured by it, it will be found that there is a very evident obstruction of the larynx remaining after the freest vomiting; while in the spasmodic croup, the breathing is generally found to be so completely restored to its natural character, that it is scarcely audible in a very short time after the emesis has been produced. (*To be continued.*)

